# Invasive Fungal Rhinosinusitis: Clinical and Surgical Factors Affecting Its Prognosis and Disease-Specific Morality

**DOI:** 10.7759/cureus.38830

**Published:** 2023-05-10

**Authors:** Fatima Shahid, Asmara Hussain, Nur Ul Ain, Muzna Mehmood Bhatti

**Affiliations:** 1 Otorhinolaryngology, Rawalpindi Medical University, Rawalpindi, PAK; 2 Otolaryngology, District Headquarter Hospital, Chakwal, PAK; 3 Plastic and Reconstructive Surgery, Holy Family Hospital, Rawalpindi, PAK; 4 Internal Medicine and Surgery, Rawalpindi Medical University, Rawalpindi, PAK

**Keywords:** intra-orbital, intracranial, disease-specific mortality, palatal involvement, mucormycosis, diabetes mellitus, mortality, rhinosinusitis, paranasal sinus, invasive fungal rhinosinusitis

## Abstract

Purpose: The purpose of this study is to discover factors that determine mortality in patients with invasive fungal rhinosinusitis.

Methods: This retrospective study included 17 patients diagnosed with invasive fungal rhinosinusitis and who had undergone treatment in our department, including both surgical and medical management between January 2020 and October 2020. There were four male patients and 13 female patients whose mean age was 46 ± 15.67 years, ranging from 20 to 70 years. All the patients were immune-compromised because of diabetes mellitus. We studied the factors affecting the mortality of patients affected with this disease; it included the extent of disease (paranasal sinus, palatal, orbital, or intracranial involvement), serum glucose level (SGL), and C-reactive protein (CRP) levels.

Results: Only one patient had paranasal sinus involvement alone, but the patient became disease-free after treatment. The disease-specific mortality rate was two (33.3%) of six patients with palatal involvement and four (50%) of eight patients with intracranial involvement where four patients did not have disease control at the time of discharge and did not follow up. The death rate in orbital involvement was 20% (three of 15 patients) and five patients with intra-orbital involvement left the hospital against medical advice.

The analysis of data demonstrated that only intracranial (p = 0.01) involvement in addition to the nasal cavity and paranasal sinus involvement had a statistically significant effect on survival rate unlike intra-orbital (p = 0.510) and palatal involvement (p = 0.171).

Conclusion: Early endoscopic nasal inspection, diagnosis, and treatment are critical for disease-specific mortality in invasive fungal rhinosinusitis since orbital or cerebral involvement is linked to a poor prognosis. Patients who come with uncontrolled diabetes and ophthalmological and palatal involvement with positive findings on nasal examination should necessitate urgent histopathological and radiological workup.

## Introduction

Invasive fungal rhinosinusitis is a type of fungal rhinosinusitis with a high mortality rate mainly affecting people with immunocompromised state such as patients suffering from diabetes mellites, AIDS, and hematological malignancies or patients with organ transplant. The most common presentation is of rhino-orbito-cerebral form following pulmonary, gastrointestinal, cutaneous, and renal invasive fungal rhinosinusitis. Causative pathogens are from the family Mucoraceae, which are *Absidia*, *Rhizopus*, and *Mucor*. In diabetic patients, the most common cause is zygomycetes. Infections produced by the fungus in the class Zygomycetes include the orders Mucorales and Entomophthorales. They usually reside in the nose through inhalation. Usually, Mucorales are killed by normal hosts' mononuclear and polymorphonuclear phagocytes through oxidative metabolites [1,2]. By both oxidative and nonoxidative mechanisms, hyperglycemia and acidosis are known to impair phagocytes' ability to move forward and kill organisms [3]. Hence, patients with diabetic ketoacidosis are more prone to rhino-cerebral invasive fungal rhinosinusitis. Multiple lines of evidence support the concept that patients with systemic acidosis have higher amounts of accessible serum iron, which is most likely due to iron release from binding proteins in the presence of acidosis [4]. The early identification and treatment of invasive fungal rhinosinusitis are critical for a good prognosis and survival. A definitive diagnosis can be made on histopathological examination by the presence of fungal hyphae in the involved tissue [5]. Four factors are critical in treatment: rapid diagnosis, the reversal of the underlying immunocompromised state (if possible), the prompt administration of the systemic antifungal agents, and urgent and aggressive surgical debridement [6].

A vast number of studies have been carried out regarding the management of invasive fungal rhinosinusitis, but prognostic tools have not been clearly defined yet. The aim of this study was to present the demographic pattern and features of this disease and to highlight the importance of early diagnosis and intervention in this disease. The impact of patient-related factors on prognosis and survival was also investigated.

## Materials and methods

This retrospective study included 17 patients diagnosed with invasive fungal rhinosinusitis, who had undergone treatment in the department of otolaryngology in Holy Family Hospital, Rawalpindi, including both surgical and medical management from January 2020 to October 2021. The study was conducted after approval from the hospital's ethical review board.

The diagnosis of invasive fungal rhinosinusitis was established by the presence of fungal hyphae on histopathological examination and characteristic magnetic resonance imaging (MRI)/computed tomography (CT) nose and paranasal sinus findings in an immunocompromised patient. The diagnostic criteria used were as follows: (1) having an underlying disease or immunocompromised conditions such as uncontrolled diabetes mellitus, with or without the use of long-acting systemic steroids; (2) fever, nasal blockage, face pain, hypoesthesia, vision loss, impaired orbital movement, or changed mental status being possible symptoms; (3) the presence of necrotic turbinate or greenish black slough in the nasal cavity with or without ophthalmoplegia, proptosis, or visual loss; (4) the presence of fungal organism on histopathological examination; and (5) the presence of heterogenous opacity and/or bony erosions on computed tomography (CT) or magnetic resonance imaging (MRI).

A complete medical history was taken from all the patients, and a thorough head and neck examination was performed. The underlying cause was discovered. An endoscopic examination was done to inspect the nasal cavity. The presence of ptosis, proptosis, chemosis, diplopia, partial or total ophthalmoplegia, vision loss, or blindness was noted and documented. Complete blood account parameters, serum glucose level (SGL), and C-reactive protein (CRP) levels were obtained. The extent of the disease was determined using CT and MRI scans.

**Figure 1:**
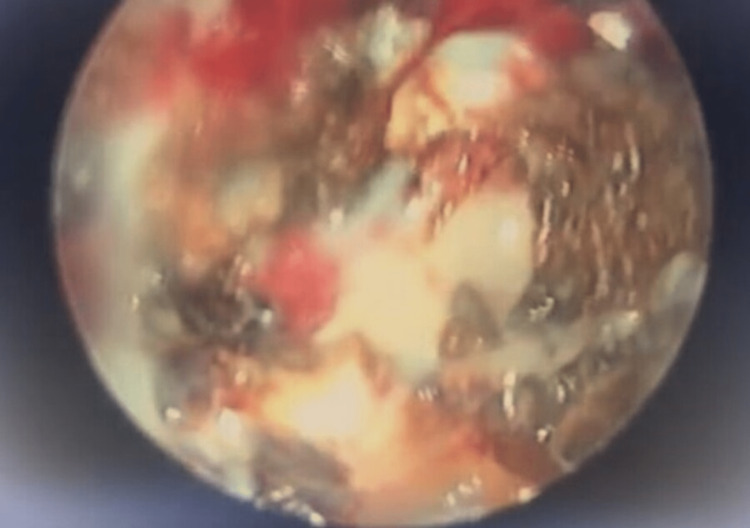
Endoscopic view of the patient with extensive rhino-orbital mucormycosis

The key parameters that were used in this study were as follows: (1) The invasive fungal rhinosinusitis involvement of anatomical areas was graded as follows: grade 1, isolated paranasal sinus involvement; grade 2, orbital and/or palate involvement; and grade 3, intracranial involvement with or without cranial nerves involvement. (2) Complete blood count, blood glucose level, and serum CRP levels were taken, and their values were used to evaluate the effect on disease-specific mortality (Figure 1).

**Figure 2:**
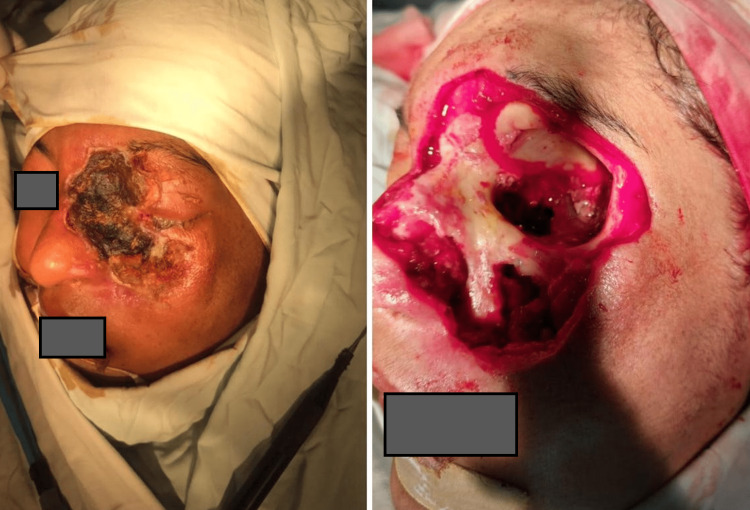
Pre- and postoperative picture of the patient with extensive rhino-orbital mucormycosis

Once diagnosis was established, amphotericin B injection (liposomal) in a dose of 1-1.5 mg/kg was started. All patients with positive nasal findings underwent urgent surgical debridement, and tissues were sent for histopathological view and tissue culture. Histopathologists examined tissue sections under a light microscope with hematoxylin and eosin (H&E), and septate and nonseptate hyphae causing angioinvasion were seen on tissue evaluation. Aggressive surgical debridement was done via endoscopic and open approach to remove all the necrotic tissue. Orbital exenteration was performed in two patients with complete visual loss (no perception of light) and ophthalmoplegia as documented by the ophthalmologist (Figure 2).

Statistical analysis was performed using Statistical Package for Social Sciences (SPSS) version 23 (IBM SPSS Statistics, Armonk, NY). Data were shown as mean ± SD for continuous variables, and the number of cases was used for categorical ones. Chi-square test was used to compare the number of cases with orbital, palatal, and intracranial involvement who had died to those who had survived with or without disease control.

## Results

**Figure 3:**
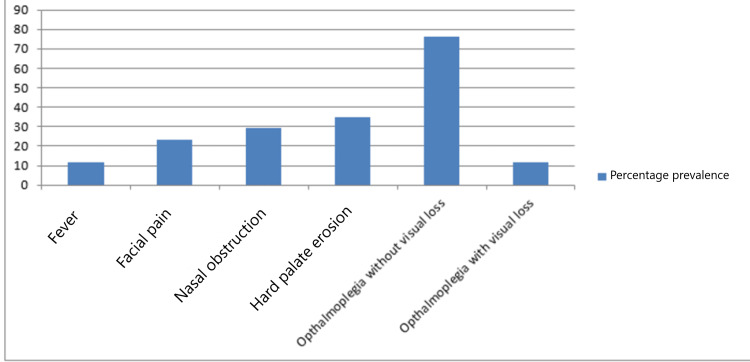
Percentage prevalence of symptoms associated with invasive fungal rhinosinusitis

In our study, four patients were male, and 13 were female. The mean age was 46 ± 15.67 years, ranging from 20 to 70 years. Symptoms at admission were fever in two (11.7%), facial pain in four (23.5%), total visual loss and ophthalmoplegia in two (11.7%), nasal obstruction in seven (29.4%), partial ophthalmoplegia without visual loss in 13 (76.4%), and wound on the hard palate in six patients (35%). Diabetes mellitus was the underlying cause in all patients with invasive fungal rhinosinusitis (Figure 3).

The sites involved on radiological examination were the orbit in 15 patients, the middle turbinate in 12, the palate in six, the nasal septum in two, and the inferior turbinate in one. Intracranial involvement was seen in eight patients (Table 1).

**Table 1:** Frequency of radiological sites of invasion PNS: paranasal sinus

Serial Number	Site Involved	Frequency
1	PNS Involvement	17 (100%)
2	Intra-orbital Involvement	15 (88.2%)
3	Intracranial Involvement	8 (47.1%)
4	Palatal Involvement	6 (35.3%)

The average blood sugar levels and CRP levels at presentation were 341 mg/dl and 41.12 mg/dl, respectively. Invasive fungus was seen in all tissue biopsies on histological analysis; however, tissue culture was only positive for fungus in four patients (23.5%). Of the total patients, four (23.5%) showed septate hyphae, and 13 (76.5%) showed aseptate hyphae. Surgical approaches used were solely endoscopic in two patients (11.7%) and open approach with maxillectomy and ethmoidectomy in 15 patients (88.3%). The orbital exenteration of involved side was done in two (11.7%) as these patients had gross. Only one patient had paranasal sinus involvement alone, and the patient became disease-free after treatment. Disease-specific mortality was in two (33.3%) of six patients with palatal involvement, in one (50%) of two patients with orbital exenteration, and in four (100%) of eight patients with intracranial involvement, and four patients did not have disease control at time of discharge and did not follow up. Death rate in orbital involvement was 20% (three of 15 patients), and five patients with intra-orbital involvement left the hospital against medical advice.

**Figure 4:**
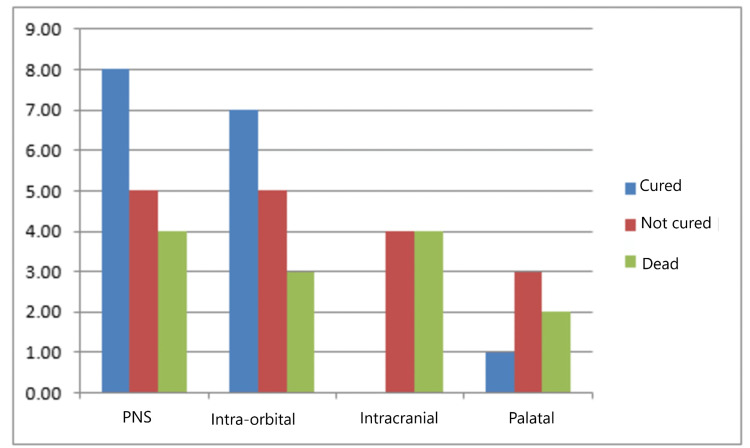
Representation of the prognosis of disease with respect to the site of invasion PNS: paranasal sinus

The analysis of data demonstrated that only intracranial (p = 0.01) involvement in addition to the nasal cavity and paranasal sinus involvement had statistically significant effect on survival rate unlike intra-orbital (p = 0.510) and palatal involvement (p = 0.171) (Figure 4).

## Discussion

In this study, we discovered that the extent of the disease was the only component that had a significant impact on disease-specific mortality in invasive fungal rhinosinusitis patients [7]. Invasive fungal rhinosinusitis is caused by opportunistic infection in immunocompromised individuals. Mucormycosis is now a term used to characterize illnesses caused by fungi belonging to the Mucorales order. *Rhizopus* species (spp.), *Mucor* spp., and *Lichtheimia* spp. (previously of the genera *Absidia* and *Mycocladus*) are the most commonly reported pathogens in invasive fungal rhinosinusitis, followed by *Cunninghamella* spp., *Apophysomyces* spp., and *Saksenaea* spp. [8,9].

The underlying diseases contributing to immunocompromised status include uncontrolled diabetes mellitus, diabetic ketoacidosis, solid organ/bone marrow transplantation, acquired immunodeficiency syndrome, prolonged corticosteroid, deferoxamine use, and high serum iron levels [10]. In diabetic patients, the most common cause is zygomycetes. Infections produced by fungus in the class Zygomycetes include the orders Mucorales and Entomophthorales. They usually reside in the nose and enter through inhalation.

Patients with elevated accessible serum iron are more susceptible to invasive fungal rhinosinusitis, according to a recently discovered key clinical characteristic. For more than two decades, researchers have known that patients taking the iron chelator deferoxamine have a significantly higher risk of invasive fungal rhinosinusitis [11].

Our study only included patients with diabetes mellitus. Presenting symptoms include fever, nasal obstruction, facial pain, orbital swelling, diplopia, visual loss, and alternation in mental status [12]. In this study, nasal obstruction was the most common presenting symptom.

**Figure 5:**
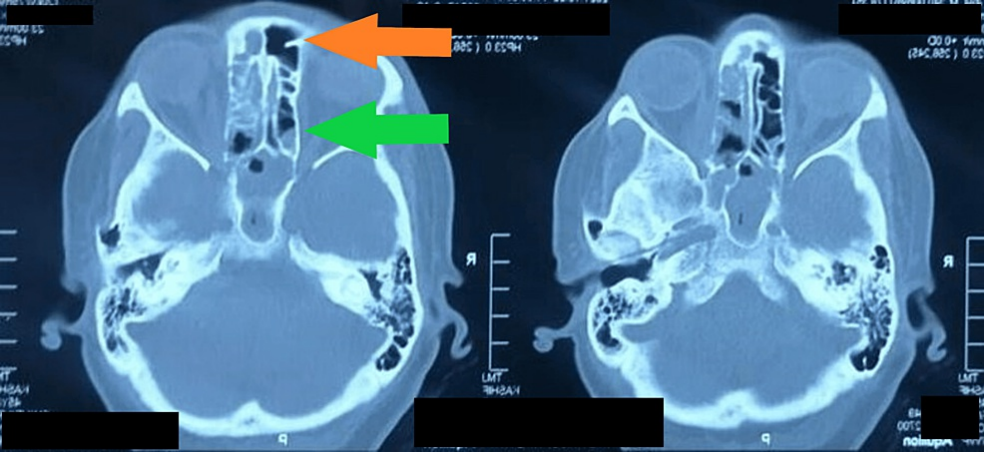
CT scan axial view showing heterogeneous opacification of anterior and posterior ethmoids and sphenoid sinuses Orange arrow, anterior ethmoid sinus; green arrow, posterior ethmoid sinus CT: computed tomography

**Figure 6:**
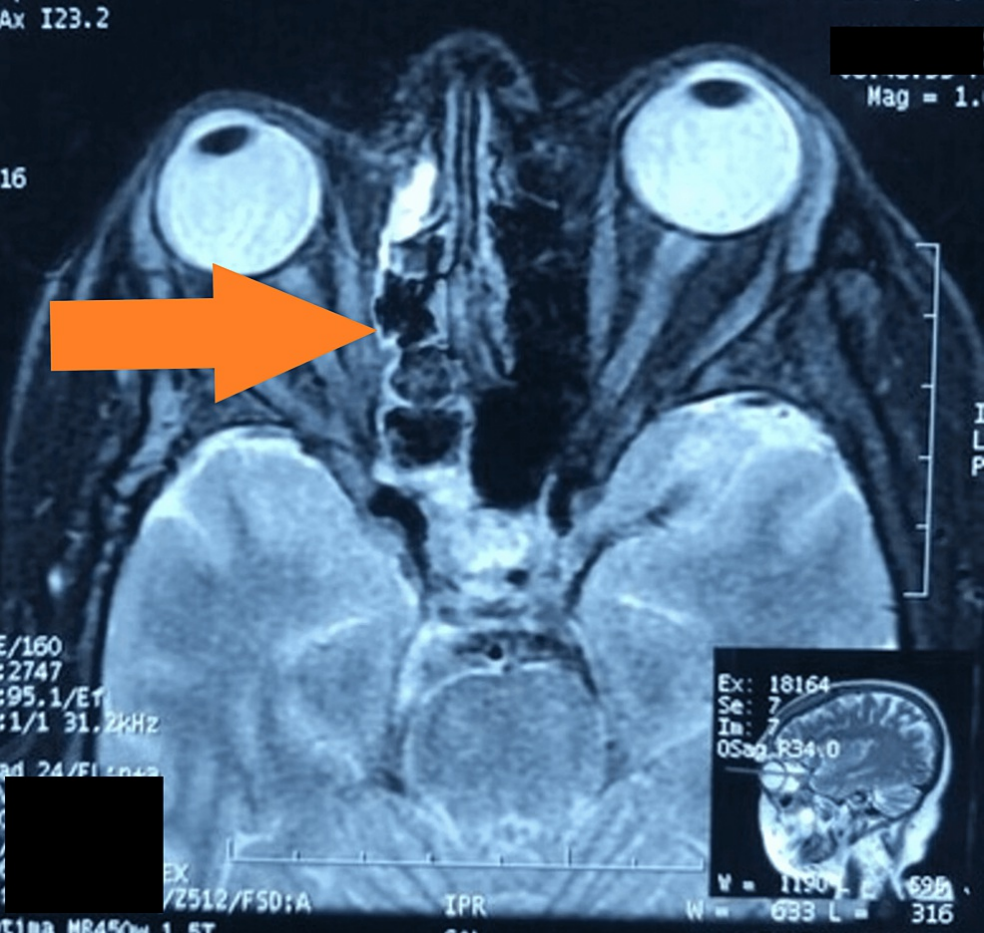
MRI T2-weighted image showing heterogeneous opacification of anterior and posterior ethmoids and sphenoids on right side of the nasal cavity MRI: magnetic resonance imaging

**Figure 7:**
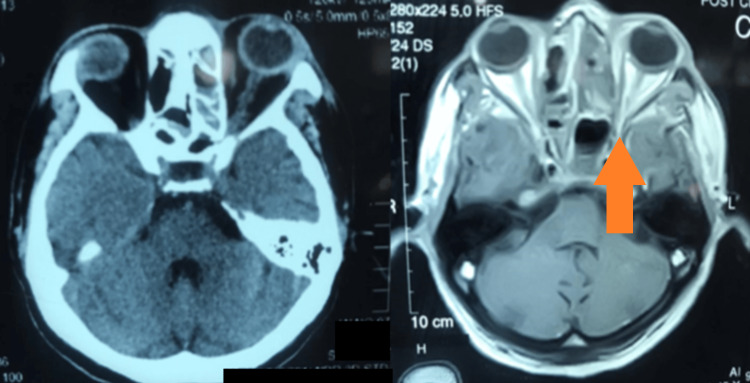
CT scan and MRI axial view of same patient showing double densities with loss of contrast enhancement on MRI T1WI Orange arrow: double densities CT, computed tomography; MRI, magnetic resonance imaging; T1WI, T1-weighted image

**Figure 8:**
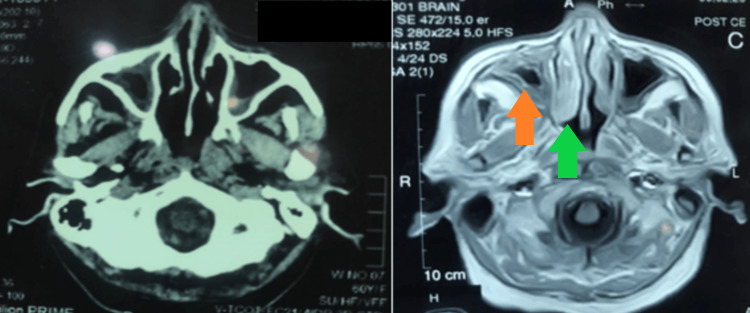
CT scan and MRI axial view showing mucormycosis Orange arrow, maxillary sinus; green arrow, interior turbinate CT, computed tomography; MRI, magnetic resonance imaging

Patients presenting with these complaints should be examined in detail with endoscopes, and samples should be sent for histopathological examination and tissue culture. Blood glucose level and CRP should be obtained. Other risk factors should be identified. Urgent radiological investigations should be done. A CT scan will show bone erosions. The presence of trace metals such as manganese, the high protein and decreased water content of the secretions, the existence of fungal hypha, or a combination of all of these traits may explain the heterogeneous appearance of these fungal sinus secretions [13,14]. MRI findings for invasive fungal rhinosinusitis include non-enhancing hypointense turbinates (also known as the "black turbinate sign"), sinus opacification, air-fluid concentration, obliteration of the nasopharyngeal planes, variable intensity within the sinuses on T1- and T2-weighted images (more likely hypointense on T2), and loss of contrast enhancement (LoCE) of the sino-nasal mucosa and extraocular muscles (Figures 5-8) [15,16].

Imaging may help, but definitive diagnosis can only be made on histopathological examination. To confirm an infection, tissue slices stained with hematoxylin and eosin (H&E), periodic acid-Schiff (PAS) stain, or Grocott's methenamine silver (GMS) stain, or all, must exhibit nonpigmented hyphae showing tissue penetration [17].

Histopathological confirmation for the diagnosis is more sensitive as compared to tissue culture [18]. In our study, all tissue biopsies demonstrated invasive fungal rhinosinusitis; however, tissue cultures were positive in only two patients.

Early identification and fast treatment options such as intravenous (IV) antifungal medication (in case of invasive fungal rhinosinusitis, IV amphotericin B injection), vigorous debridement, and managing the underlying illnesses are critical. The prognosis for medical treatment alone is worse than for medical treatment combined with surgical debridement [19]. Radical surgical procedures should be performed immediately if diagnosis is confirmed by tissue biopsy or culture. Surgical approaches used were solely endoscopic in two patients (11.7%) and open approach with maxillectomy and ethmoidectomy in 15 patients (88.3%). Orbital exenteration of the involved side was done in two (11.7%). Nair and Dave pointed out in their study that transcutaneous retrobulbar injection of amphotericin B can be considered as an option in certain cases of limited orbital disease or when there is orbital mucormycosis where debridement or exenteration is not indicated [20].

Disease confined to the paranasal sinuses may have a favorable prognosis, whereas cerebral or orbital involvement is linked to a higher risk of death [21]. A delayed diagnosis can lead to a far more serious illness. The main cause of disease-related mortality could be intracranial involvement. The main gateways for infection transmission into the orbit are the lamina papyracea or the roof of the maxillary sinus [22]. Intracranial spread can be accessed by the cribriform plate or orbital apex. According to Peterson et al., those who had orbital involvement had a greater mortality rate than those who did not have orbital involvement [19].

Kulkarni et al. describe that prothrombotic state created by COVID-19 is a risk factor for developing invasive mucormycosis [23]. A study conducted during the COVID-19 pandemic recommended to look for bony erosions in case of any sinusitis, particularly at the bony maxillary walls and the turbinates. It further suggests to look at the intra-orbital compartment thoroughly even in the absence of bony erosion [24]. A comprehensive clinical-radiological classification system for patients with maxillofacial mucormycosis has been suggested by Tomar et al. [25].

When comparing intracranial involvements to isolated nasal cavity/paranasal sinus involvement or intra-orbital involvement in our study, univariate analysis revealed that intracranial involvement was associated with the disease-specific mortality. Furthermore, the severity of the condition was the only independent variable that had a significant impact on disease-specific mortality rates. To stop the infection and improve prognosis, early identification and aggressive surgical debridement are required.

All-cause mortality rates for invasive fungal rhinosinusitis range from 40% to 80% with varying rates depending on underlying conditions and sites of infection [21]. Hence, timely diagnosis is very important for the survival of such patients.

## Conclusions

In invasive fungal rhinosinusitis patients, early identification and treatment are critical because delay in diagnosis can lead to orbital and intracranial involvement. This can further increase the risk of disease-specific mortality in such patients. Early endoscopic nasal inspection, diagnosis, and treatment are critical for disease-specific mortality in invasive fungal rhinosinusitis since orbital or cerebral involvement is linked to a poor prognosis. Patients who come with uncontrolled diabetes, ophthalmological findings, and palatal findings and with positive findings on nasal examination should necessitate urgent histopathological and radiological workup.
